# Quantitative Analysis of Macular Inner Retinal Layer Using Swept-Source Optical Coherence Tomography in Patients with Optic Tract Syndrome

**DOI:** 10.1155/2017/3596587

**Published:** 2017-06-28

**Authors:** Katsutoshi Goto, Atsushi Miki, Tsutomu Yamashita, Syunsuke Araki, Go Takizawa, Kenichi Mizukawa, Yoshiaki Ieki, Junichi Kiryu

**Affiliations:** ^1^Department of Ophthalmology, Kawasaki Medical School, Kurashiki City, Okayama, Japan; ^2^Department of Sensory Science, Faculty of Health Science and Technology, Kawasaki University of Medical Welfare, Kurashiki City, Okayama, Japan; ^3^Graduate School of Health Science and Technology, Kawasaki University of Medical Welfare, Kurashiki City, Okayama, Japan; ^4^Shirai Eye Hospital, Mitoyo City, Kagawa, Japan

## Abstract

**Objective:**

To evaluate macular inner retinal layers using swept-source optical coherence tomography (SS-OCT) in patients with homonymous hemianopia due to optic tract syndrome (OTS).

**Methods:**

Sixteen eyes of 8 patients with OTS were studied. The macular retinal nerve fiber layer (mRNFL), ganglion cell layer and inner plexiform layer (GCL + IPL), and mRNFL and GCL + IPL (GCC) were measured by SS-OCT (DRI OCT-1 Atlantis®).The scanned area was divided into eight regions and two hemiretinae. Each retinal thickness of the OTS group was compared with that of the 25 control subjects.

**Results:**

The GCC thickness in the ipsilateral eyes was significantly reduced in all regions, although predominant thinning of the GCC in the contralateral eyes was found in the nasal region. The GCC + IPL thickness was preferentially reduced at the temporal regions in the ipsilateral eyes and at the nasal regions in the contralateral eyes. The reduction rate of the GCL + IPL thickness was 29.6% at the temporal hemiretina in the ipsilateral eyes and 35.2% at the nasal hemiretina in the contralateral eyes.

**Conclusion:**

We found preferential loss of the GCC + IPL thickness corresponding to the hemifield defects in each eye. Quantitative analysis by SS-OCT is capable of detecting the characteristic RGC loss due to OTS.

## 1. Background

Optic tract syndrome (OTS) is caused by a unilateral optic tract lesion, which leads to homonymous hemianopia, relative afferent pupillary defect (RAPD) in the contralateral eyes, and characteristic hemianopic optic atrophy in both eyes [1–3]. Hemianopic optic atrophy represents hour glass atrophy in the ipsilateral eyes and band atrophy (BA) in the contralateral eyes [4, 5]. The presence of hemianopic optic atrophy in both eyes and RAPD in the contralateral eyes are useful in the different diagnosis of OTS.

Optical coherence tomography (OCT) is capable of quantifying the circumpapillary retinal nerve fiber layer (cpRNFL) and macular inner retinal layer. OCT may be useful not only in patients with retinal or optic nerve disorders but also in those with cerebral lesions [6]. Some investigators have reported that the cpRNFL and ganglion cell complex (GCC) thicknesses using time-domain and spectral-domain (SD) OCT in OTS patients were significantly reduced in both eyes [7–9].

To the best of our knowledge, there have been no reports of a quantitative analysis of the macular inner retinal layer thickness using swept-source OCT (SS-OCT) in OTS patients. The macular inner retinal thickness measurements are considered to be sensitive for detecting the retinal ganglion cell (RGC) loss caused by an optic tract lesion, because more than 50% of RGCs are distributed within a diameter of 4.5 mm in the macula [10]. Thus, the purpose of the present study was to evaluate characteristic hemianopic optic atrophy using the macular inner retinal layer thickness measurements by SS-OCT in patients with OTS.

## 2. Methods

Patients with traumatic OTS due to a traffic accident diagnosed by an ophthalmic examination agreed to participate in this study at the Department of Ophthalmology in Kawasaki Medical School Hospital. Normal subjects recruited as an age-matched control group were also enrolled. The study protocol adhered to the tenets of the Declaration of Helsinki and was approved by the institutional review board of Kawasaki Medical School. All patients underwent ocular examinations including measurements of the best corrected visual acuity, a slit-lamp examination, intraocular pressure measured with Goldmann applanation tonometry, Goldmann perimetry (Haag-Streit AG, Bern, Switzerland), funduscopy, fundus photography, and SS-OCT. OTS patients also underwent computed tomography (CT) and magnetic resonance imaging (MRI). The RAPD in patients with OTS was observed in swinging flash light test by experienced neuro-ophthalmologist (A.M.) and/or RAPDx® (Konan Medical, Inc., Irvine, CA, USA) automated pupillography. The diagnosis of OTS was based on the presence of homonymous hemianopia and the RAPD on the eyes with temporal visual field defect examined using swinging flash light test and/or RAPDx® pupillography. Also, special attention was paid to exclude cerebral infarction, cerebral hemorrhage, brain tumor, and congenital brain lesions affecting the retrochiasmal visual pathway using CT and MRI. The other inclusion criteria for OTS were as follows: best corrected visual acuity 20/20 or better, range of spherical refractive power from −5.75 diopters (D) to +2.75D, cylinder refractive power within ±3.00D, and intraocular pressure < 22 mmHg. The exclusion criteria were as follows: history of intraocular surgery, retinal diseases, optic nerve diseases, such as glaucoma, and dense cataracts affecting the quality of the SS-OCT images.

The inclusion criteria for the normal control subjects were as follows: best corrected visual acuity 20/20 or better, range of spherical refractive power from −5.75 diopters (D) to +2.75D, cylinder refractive power within ±3.00D, intraocular pressure < 22 mmHg and no history of intraocular surgery or trauma, retinal diseases including diabetic retinopathy, optic nerve diseases, such as glaucoma, or other any disease affecting the visual field. Patients with dense cataracts affecting the quality of the SS-OCT images were excluded. Normal subjects underwent evaluations with a Humphrey field analyzer® (Carl Zeiss Meditec., Dublin, CA, USA) using the central 30-2 Swedish Interactive Threshold Algorithm (SITA) program. A normal visual field was defined as the absence of any cluster of at least three points with *P* < 5%, one point *P* < 0.5%, or 1% on the pattern deviation probability plot, excluding the two points above and below the blind spot and within the normal limits in the glaucoma hemifield test and the 95% confidence interval. Reliable visual field results obtained with the Humphrey field analyzer were defined as those with a fixation loss of less than 20% and false positive and false negative errors of less than 20%.

### 2.1. Macular Inner Retinal Layer Thickness Measured Using SS-OCT

SS-OCT examinations were performed using DRI OCT-1 Atlantis® (Topcon Corp., Tokyo, Japan). The specification of this SS-OCT was such that the light source was a 1050 nm wavelength tunable laser with a spectrum band width of 100 nm, axial resolution of 8.0 *μ*m, a scan/second of 100,000, and a focus range from −33.00D to +40.00D. The SS-OCT software program version 9.14 was for the data analysis.

**Figure 1:**
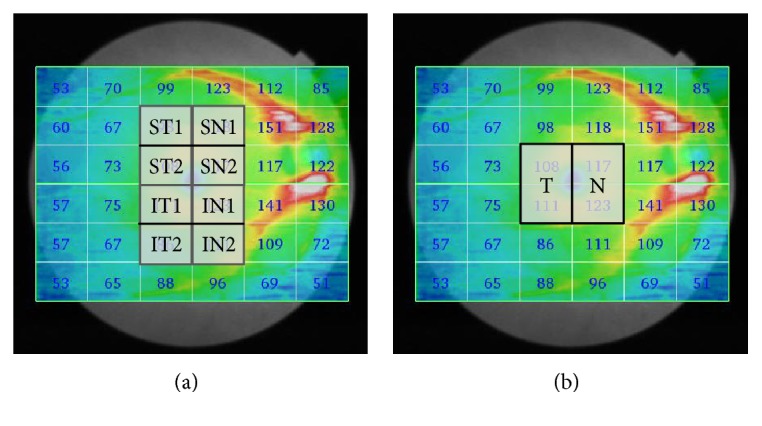
Division of the measurement region in the macular inner retinal layer thicknesses obtained using SS-OCT. The scanning protocol used was 3D macula with a scan density of 512 × 256 covering a 12 × 9 mm^2^ area. The data analysis used was 36 rectangular patterns and one check size measuring 2 × 1.5 mm. (a) The 4 × 6 mm^2^ area around the fovea was divided into eight regions: superotemporal (ST1, ST2), inferotemporal (IT1, IT2), superonasal (SN1, SN2), and inferonasal (IN1, IN2). (b) The 4 × 3 mm^2^ area around the fovea was divided into two hemiretinae along the vertical meridian: temporal (T), nasal (N). SS-OCT: swept-source optical coherence tomography.

SS-OCT examinations were performed two times on the same day of the visual field test by experienced technicians, and the most reliable data were selected. Image quality scores < 40 or with segmentation errors were excluded. The scanning protocol used was 3D macula with a scan density of 512 × 256 covering a 12 × 9 mm^2^ area. The data analysis used was 36 rectangular patterns and one check size measuring 2 × 1.5 mm. The 4 × 6 mm^2^ area around the fovea was divided into eight regions as follows: superotemporal (ST1, ST2), inferotemporal (IT1, IT2), superonasal (SN1, SN2), and inferonasal (IN1, IN2). The 4 × 3 mm^2^ area around the fovea was divided into two hemiretinae; temporal (T), nasal (N) (Figure 1()). The macular inner retinal layer thicknesses measured were as follows: the macular retinal nerve fiber layer (mRNFL), ganglion cell layer and inner plexiform layer (GCL + IPL), mRNFL and GCL + IPL (GCC). Each inner retinal layer thickness in the OTS group was compared with the normal control group. The eyes on the same side of the optic tract lesion were defined as the ipsilateral eyes and the eyes on the opposite side as the contralateral eyes.

### 2.2. Statistical Analysis

Statistical analyses were performed using the Statistical Package for Social Science software package version 22.0 (SPSS, IBM, Tokyo, Japan). The two-sample Student's *t*-test and Fisher's exact test were used to detect differences in age and gender between normal control subjects and OTS patients. Each inner retinal thickness and refractive error in ipsilateral eyes and contralateral eyes were compared with those in normal control subjects using the generalized linear mixed model. The dependent variable was the inner retinal thickness and refractive error. The fixed factor was ipsilateral eye, contralateral eye, or normal eye, and the variable factor was the eye number (one to 41). A statistically significant difference was defined by a value of *P* < 5%.

## 3. Results

Eight patients with traumatic OTS and 25 normal control subjects were included in this study. The clinical data for OTS patients is shown in Table 1. OTS patients included seven males and one female with an average age of 40.4 ± 15.5 years. Their average spherical equivalent was −1.81 ± 2.38D in the ipsilateral eyes and −1.56 ± 2.03D in the contralateral eyes, and the average disease duration from the traffic accident to examination was 115.5 ± 121.7 months. All patients had 20/20 or better visual acuity. Of the eight patients with OTS, six had incomplete homonymous hemianopia and two had complete homonymous hemianopia. An RAPD was always detected in the eyes with temporal hemifield defects by subjective evaluation and/or RAPDx® pupillography. The average RAPD amplitude score in the patients tested using RAPDx® pupillography was 0.49 ± 0.18 log units.

Normal control subjects included 16 males and 9 females with an average age of 44.0 ± 10.4 years (21 to 59 years). Their average refractive error in spherical equivalents was −2.45 ± 2.09D (+0.50D to −5.25D). There was no significant difference in the age or refractive error between OTS eyes and normal control eyes (Table 2).

Table 3 shows each inner retinal layer thickness using SS-OCT in the OTS and normal control groups. The GCC thickness in the ipsilateral eyes of the OTS group was significantly reduced in all regions compared with that of the normal control group. In the contralateral eyes, the GCC thickness was predominantly reduced in all the nasal regions in the OTS group than in the normal control group. The GCL + IPL thickness in the ipsilateral eyes was preferentially reduced in the temporal regions. In the contralateral eyes, the GCL + IPL thickness was preferentially reduced in the nasal regions. The mRNFL thickness was also significantly reduced in each eye, and the thinning pattern was generally similar to that of GCC thicknesses.

## 4. Discussion

Our results indicated that the macular inner retinal layer thickness in OTS patients was significantly reduced compared with that of the normal control eyes. This reduction was especially pronounced in the nasal hemiretina of the contralateral eyes and in the temporal hemiretina of the ipsilateral eyes.

In previous reports using SD-OCT in OTS patients, Kanamori et al. [9] reported that the GCC and cpRNFL thicknesses were significantly reduced in four patients with OTS. As a result, the GCC analysis revealed that characteristic thinning of the GCC in the temporal hemiretina of the ipsilateral eyes and in the nasal hemiretina of the contralateral eyes corresponded to the optic tract lesions. Monteiro et al. [11] reported that SD-OCT showed thinning of the mRNFL and GCL and thickening of the inner nuclear layer in a patient with long-standing OTS.

To the best of our knowledge, this is the first report of a detailed quantitative analysis of the macular inner retinal layer thickness using a grid analysis by SS-OCT in OTS patients. We found that each area of analysis using a rectangular pattern is capable of detecting characteristic RGC loss along foveal vertical meridian due to OTS. Regarding the reports of comparison of the diagnostic abilities of SD-OCT and SS-OCT, detection accuracy of thinning of the macular inner retinal layer using SS-OCT in glaucomatous eyes was comparable to that of SD-OCT [12, 13]. In addition, SS-OCT has clear advantages over other techniques in terms of its ultrahigh-speed imaging, high sensitivity, high penetration of fundus tissue, and speckle noise reduction. SS-OCT may be useful for the accurate measurement in patients with fixation loss or unstable head position due to visual field defects.

We found that the GCL + IPL thickness was preferentially reduced at the temporal regions in the ipsilateral eyes and at the nasal regions in the contralateral eyes. These thinning regions showed that the preferential loss along the vertical meridian corresponds to the hemi visual field defects in each eye. The GCL + IPL thickness measurement is useful for detecting the characteristic RGC loss caused by optic tract lesions. The GCC and mRNFL thicknesses in the ipsilateral eyes were significantly reduced in all regions, although those were preferentially reduced in the nasal regions in the contralateral eyes. The uncrossing fibers from the temporal hemiretina arrive at the optic disc through the nasal hemiretina after crossing the foveal vertical meridian. Therefore, the nasal hemiretina measured by SS-OCT must include uncrossing fibers from the temporal hemiretina. The projection pattern of damaged uncrossing fibers results in a significant loss in both hemiretinae. Therefore, the GCC and mRNFL thicknesses in the ipsilateral eyes could be reduced even in the nasal regions, as well as the temporal regions, due to the uncrossing fiber loss.

There are several limitations associated with the present study. First, this study included a small number of patients. Accordingly, a subgroup analysis could not be performed according to the pattern of visual field loss. Second, the disease duration was highly variable among patients. Third, the accurate time of retrograde degeneration of RGCs to plateau is unclear because we did not analyze its time course. Further longitudinal analyses with a larger sample size are necessary to fully elucidate the inner retinal layer loss in patients with OTS.

In conclusion, we demonstrated via SS-OCT that characteristic hemianopic optic atrophy corresponds to the side of the optic tract lesion. A grid analysis of the macular inner retinal layer thickness is useful for diagnosing OTS, because SS-OCT is capable of detecting the characteristic RGC loss due to OTS. SS-OCT also has a potential to support the diagnosis of homonymous hemianopia caused by an optic tract lesion in patients in whom subjective examinations, including visual field tests, are impossible.

## Figures and Tables

**Table 1:** The clinical data for patients with OTS.

Patient number	Age (yr)	Gender	Refractive error (D) R/L	Optic tract lesion side	Disease duration (months)	Hemianopic side
1	41	M	0.25/0.25	L	249	R
2	59	F	−3.75/−5.25	R	108	L
3	39	M	0.00/+0.50	R	53	L
4	21	M	−0.25/−0.50	R	4	L
5	59	M	0.00/−0.25	L	4	R
6	19	M	−0.50/−0.75	L	21	R
7	34	M	−5.00/−4.25	R	153	L
8	51	M	−3.50/−4.00	R	332	L

OTS: optic tract syndrome; M: male; F: female; D: diopters; R: right; L: left.

**Table 2:** Demographic characteristics of normal control subjects and patients with OTS.

	OTS patients (*n* = 8)		Normal control subjects (*n* = 25)	
	Ipsilateral eyes	Contralateral eyes	Normal eyes	*P* values
Age (years)	40.4 ± 15.5		44.0 ± 10.4	0.457
Gender (M : F)	7 : 1		16 : 9	0.382
Refractive error (D)	−1.81 ± 2.38	−1.56 ± 2.03	−2.45 ± 2.09	1.000/0.941

OTS: optic tract syndrome; M: male; F: female; D: diopters.

**Table 3:** Inner retinal layer thicknesses obtained using SS-OCT in normal controls and patients with OTS.

	Regions	OTS		Normal eyes	*P* values	
		Ipsilateral eyes	Contralateral eyes		Ipsilateral eyes versus normal eyes	Contralateral eyes versus normal eyes
GCC (*μ*m)	ST1	^∗∗^68.1 ± 5.2 (31.0%)	93.8 ± 6.9 (5.0%)	98.7 ± 6.6	<0.001	0.191
	ST2	^∗∗^68.8 ± 11.6 (35.8%)	101.1 ± 6.7 (5.6%)	107.2 ± 7.1	<0.001	0.219
	IT1	^∗∗^74.9 ± 14.8 (33.2%)	103.8 ± 9.1 (7.4%)	112.0 ± 6.6	<0.001	0.095
	IT2	^∗∗^70.0 ± 9.1 (25.6%)	87.9 ± 11.8 (6.6%)	94.0 ± 7.3	<0.001	0.260
	SN1	^∗∗^88.0 ± 8.4 (24.5%)	^∗∗^93.5 ± 12.0 (19.8%)	116.5 ± 9.0	<0.001	<0.001
	SN2	^∗^107.3 ± 6.7 (10.3%)	^∗∗^78.0 ± 19.4 (34.8%)	119.6 ± 8.3	0.026	<0.001
	IN1	^∗∗^105.8 ± 10.6 (13.1%)	^∗∗^83.9 ± 16.7 (31.0%)	121.6 ± 7.7	0.002	<0.001
	IN2	^∗∗^86.0 ± 13.1 (24.8%)	^∗∗^93.5 ± 13.3 (18.2%)	114.3 ± 11.7	<0.001	0.001
	T	^∗∗^71.8 ± 13.2 (34.5%)	102.4 ± 7.6 (6.5%)	109.6 ± 6.6	<0.001	0.110
	N	^∗∗^106.5 ± 8.5 (11.7%)	^∗∗^80.9 ± 18.0 (32.9%)	120.6 ± 7.8	<0.001	0.006

GCL + IPL (*μ*m)	ST1	^∗∗^49.5 ± 6.6 (20.4%)	59.5 ± 3.7 (4.3%)	62.2 ± 5.8	<0.001	0.714
	ST2	^∗∗^57.0 ± 7.7 (31.6%)	78.4 ± 4.7 (6.0%)	83.4 ± 6.5	<0.001	0.199
	IT1	^∗∗^61.1 ± 8.7 (27.7%)	79.0 ± 6.1 (6.5%)	84.5 ± 6.0	<0.001	0.138
	IT2	50.0 ± 4.7 (8.4%)	52.6 ± 5.5 (3.6%)	54.6 ± 6.9	0.241	1.000
	SN1	57.3 ± 5.4 (7.3%)	^∗∗^47.1 ± 6.4 (23.7%)	61.8 ± 4.7	0.115	<0.001
	SN2	80.9 ± 5.9 (6.5%)	^∗∗^55.4 ± 15.0 (36.0%)	86.5 ± 6.5	0.345	<0.001
	IN1	78.8 ± 7.9 (7.7%)	^∗∗^56.0 ± 14.3 (34.3%)	85.3 ± 6.1	0.197	<0.001
	IN2	51.5 ± 4.5 (3.6%)	^∗∗^43.0 ± 6.8 (19.5%)	53.4 ± 5.7	1.000	<0.001
	T	^∗∗^59.1 ± 8.1 (29.6%)	78.7 ± 5.2 (6.3%)	83.9 ± 6.0	<0.001	0.114
	N	79.8 ± 6.7 (7.1%)	^∗∗^55.7 ± 14.6 (35.2%)	85.9 ± 6.1	0.205	<0.001

mRNFL (*μ*m)	ST1	^∗∗^18.5 ± 8.3 (49.6%)	34.6 ± 5.0 (5.7%)	36.7 ± 3.1	<0.001	0.602
	ST2	^∗∗^11.8 ± 6.5 (51.6%)	22.6 ± 3.8 (6.9%)	24.3 ± 2.5	<0.001	0.905
	IT1	^∗∗^13.5 ± 7.1 (50.2%)	24.9 ± 4.8 (8.2%)	27.1 ± 2.4	<0.001	0.586
	IT2	^∗∗^19.9 ± 10.1 (49.4%)	35.4 ± 7.1 (9.9%)	39.3 ± 3.6	<0.001	0.389
	SN1	^∗∗^31.0 ± 6.0 (42.9%)	^∗^46.4 ± 8.7 (14.5%)	54.3 ± 6.7	<0.001	0.023
	SN2	^∗∗^26.3 ± 1.6 (19.5%)	^∗∗^22.8 ± 6.8 (30.3%)	32.6 ± 3.9	0.003	<0.001
	IN1	^∗∗^27.0 ± 3.1 (25.1%)	^∗∗^27.9 ± 3.8 (22.7%)	36.0 ± 4.2	<0.001	<0.001
	IN2	^∗∗^34.5 ± 12.7 (43.5%)	50.4 ± 14.1 (17.5%)	61.1 ± 11.2	<0.001	0.120
	T	^∗∗^12.6 ± 6.6 (50.9%)	23.8 ± 3.9 (7.5%)	25.7 ± 1.8	<0.001	0.383
	N	^∗∗^26.6 ± 2.3 (22.5%)	^∗∗^25.3 ± 5.0 (26.3%)	33.0 ± 7.6	<0.001	<0.001

SS-OCT: swept-source optical coherence tomography; OTS: optic tract syndrome; GCC: ganglion cell complex; GCL + IPL: ganglon cell layer and inner plexiform layer; mRNFL: macular retinal nerve fiber layer; ST: superotemporal; IT: inferotemporal; SN: superonasal; IN: inferotemporal; T: temporal; N: nasal, ^∗^*P* < 0.05, ^∗∗^*P* < 0.01: generalized linear mixed model; OTS eyes compared with normal control eyes; (%): percentage of decrease in OTS eyes compared with normal control eyes.
